# The complete chloroplast genome of *Agave sisalana*

**DOI:** 10.1080/23802359.2021.1935345

**Published:** 2021-06-04

**Authors:** Xinli Yang, Xing Huang, Shibei Tan, Tao Chen, Xu Qin, Helong Chen, Jingen Xi, Kexian Yi

**Affiliations:** a College of Tropical Crops, Hainan University, Haikou, PR China; b Environment and Plant Protection Institute, Chinese Academy of Tropical Agricultural Sciences, Haikou, PR China; c Key Laboratory of Integrated Pest Management on Tropical Crops, Ministry of Agriculture and Rural Affairs, Haikou, PR China; d Hainan Key Laboratory for Monitoring and Control of Tropical Agricultural Pests, Haikou, PR China; e Guangxi Subtropical Crops Research Institute, Nanning, PR China

**Keywords:** *Agave sisalana*, chloroplast genome, phylogenetic tree

## Abstract

*Agave sisalana* is one of the Agave cultivars for sisal fiber production around the tropical areas of the world. In the present study, we successfully sequenced and assembled its chloroplast genome. The full size of the genome is 157,268 bp with a GC content at 37.85%. The genome is constructed with a large single copy region (LSC, 85,894 bp), a pair of inverted repeat regions (IR, 26,573 bp) and a small single copy region (SSC, 18,228 bp). Besides, 86 protein-coding genes, 38 tRNAs and 8 rRNAs are annotated on the chloroplast genome. Phylogenetic result reveals that *A. sisalana* is closely related with *A. americana* and *A*. H11648.

Agave plants has been widely cultivated for the production of food, beverage, fiber and medicine around the world (Huang et al. 2019). Among the 166 cultivated Agave species, *Agave sisalana* is commonly used for sisal fiber production (Gil-Vega et al. 2006; Huang et al. 2018). As an important kind of natural fibers, sisal has several properties of high strength, tough texture and friction resistance, which has been widely used as high strength industrial materials for automotive, navigation, papermaking, etc. (Li et al. 2000). Agave plants are the native species in the arid and semi-arid regions in Mexico, which leads to their extreme ecological adaptation (Borland et al. 2009). The stability of chloroplast is susceptible to environmental stresses. Several studies have been reported to reveal the mechanism of heat and drought tolerance in Agave plants (Luján et al. 2009; Sarwar et al. 2019). However, few reports are related to other stresses, such as cold and lead stresses, even if they are harmful to plant chloroplast (Zhou et al. 2017; Trentmann et al. 2020). Besides, the systematic position and phylogenetic relationship of *A. sisalana* are still ambiguous at chloroplast (cp) genome level. We hence sequenced the cp genome of *Agave sisalana*, which could benefit future studies related to Agave chloroplast.

*A. sisalana* leaves were collected from the germplasm garden (22.90°N, 108.33°E) of Guangxi Subtropical Crops Research Institute, Nanning, China. The specimen was stored in Herbarium of Environment and Plant Protection Institute, Chinese Academy of Tropical Agricultural Sciences (EPPI-jm2020011). Total DNA sample was isolated by the modified CTAB method and then sent to Biozeron Biotech (Shanghai, China) for sequencing by Illumina HiSeq 2500 platform, which generated 6.0 Gb raw data (Doyle and Doyle 1987). The raw data was deposited to SRA under the accession number of PRJNA705409. The NOVOPlasty and GapCloser software were used for cp genome assembly and gap filling, respectively (Luo et al. 2012; Dierckxsens et al. 2017). We further selected the DOGMA software for genome annotation and the Geneiousv11.0.3 software for correction (Wyman et al. 2004; Kearse et al. 2012). The full cp genome sequence of *A. sisalana* was uploaded to GenBank under an accession number of MW540497.

The total length of *A. sisalana* cp genome is 157,268 with a GC content at 37.85%. The genome is constructed with a large single copy region (LSC, 85,894 bp), a pair of inverted repeat regions (IR, 26,573 bp) and a small single copy region (SSC, 18,228 bp). Besides, 86 protein-coding genes, 38 tRNAs and 8 rRNAs are annotated on the cp genome.

The cp genome sequences of 27 species were selected in phylogenetic analysis. Among these, 24 species were from Agavoideae and 3 other species were selected as outgroup, including *Albuca kirkii*, *Nolina atopocarpa* and *Oziroe biflora* (McKain et al. 2016; Lee et al. 2019; Jin et al. 2020). The alignment of the long sequences was carried out by the MAFFT software (Katoh and Standley 2013). The phylogenetic tree was constructed by the Maximum Likelihood method with 1000 bootstrap replicates in MEGA7 software (Kumar et al. 2016). The result revealed that *A. sisalana* is closely related with *A. americana*, *A. angustifolia* and *A*. H11648 (Figure 1). *A*. H11648 is an interspecific hybrid from *A. angustifolia* and *A. amaniensis*, which is also a widely cultivated *Agave* species for fiber production (Huang et al. 2019). This study would contribute to reveal the mechanism of stress tolerance related to chloroplast in *A. sisalana*.

**Figure 1.**
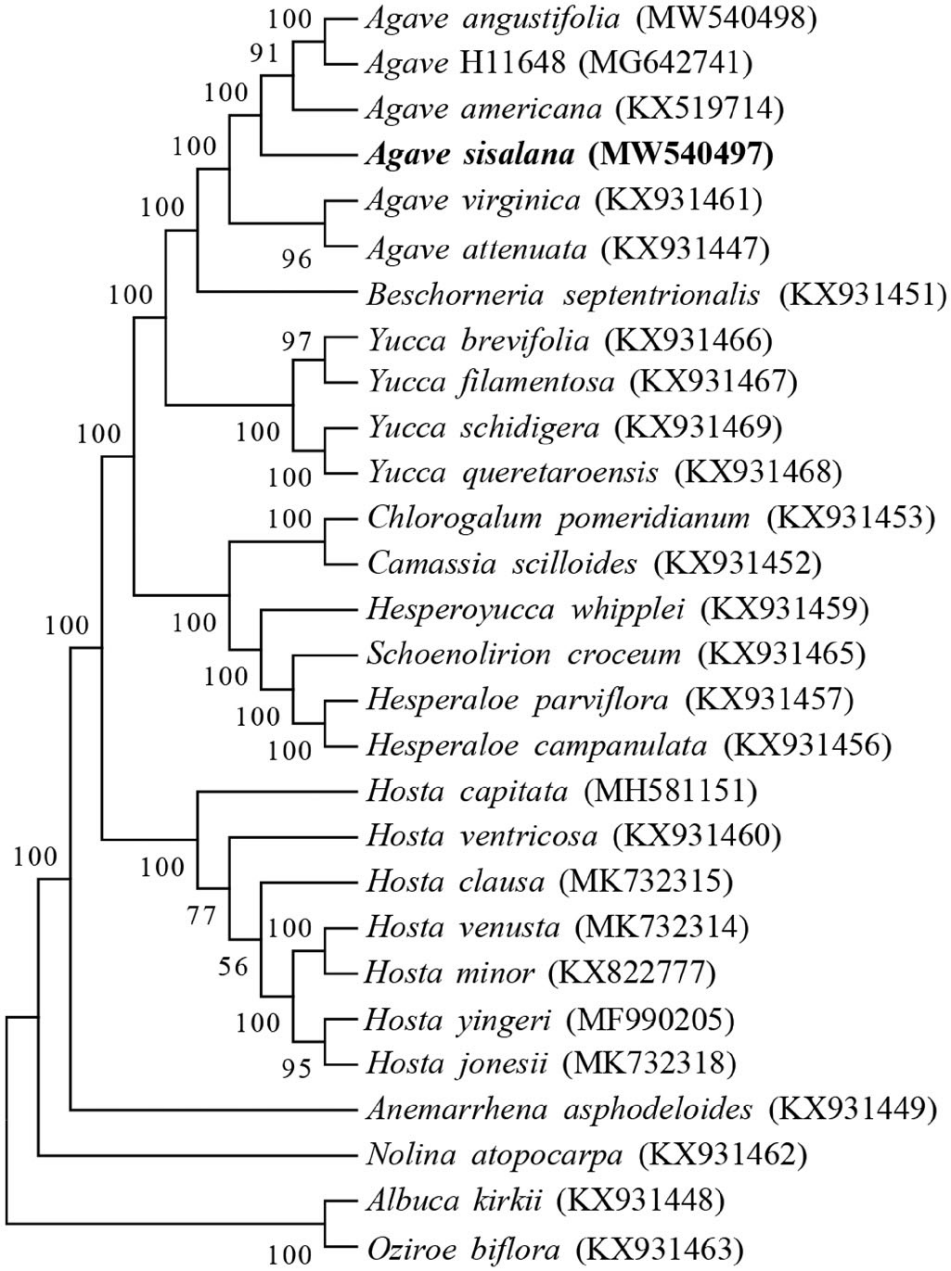
Phylogenetic tree of 28 chloroplast genomes.

## Data Availability

The data that support the findings of this study are fully available in SRA (https://www.ncbi.nlm.nih.gov/sra/?term=PRJNA705409) and GenBank (https://www.ncbi.nlm.nih.gov/nuccore/MW540497).
